# The Efficacy of the Ribonucleotide Reductase Inhibitor Didox in Preclinical Models of AML

**DOI:** 10.1371/journal.pone.0112619

**Published:** 2014-11-17

**Authors:** Guerry J. Cook, David L. Caudell, Howard L. Elford, Timothy S. Pardee

**Affiliations:** 1 Wake Forest University Health Sciences, Department of Internal Medicine, Section on Hematology and Oncology, Winston-Salem, North Carolina, United States of America; 2 Department of Pathology, Section of Comparative Medicine, Wake Forest University Health Sciences, Winston-Salem, North Carolina, United States of America; 3 Molecules for Health, Richmond, Virginia, United States of America; 4 Wake Forest University Comprehensive Cancer Center, Winston-Salem, North Carolina, United States of America; European Institute of Oncology, Italy

## Abstract

Acute Myeloid Leukemia (AML) is an aggressive malignancy which leads to marrow failure, and ultimately death. There is a desperate need for new therapeutics for these patients. Ribonucleotide reductase (RR) is the rate limiting enzyme in DNA synthesis. Didox (3,4-Dihydroxybenzohydroxamic acid) is a novel RR inhibitor noted to be more potent than hydroxyurea. In this report we detail the activity and toxicity of Didox in preclinical models of AML. RR was present in all AML cell lines and primary patient samples tested. Didox was active against all human and murine AML lines tested with IC_50_ values in the low micromolar range (mean IC_50_ 37 µM [range 25.89–52.70 µM]). It was active against primary patient samples at concentrations that did not affect normal hematopoietic stem cells (HSCs). Didox exposure resulted in DNA damage and p53 induction culminating in apoptosis. In syngeneic, therapy-resistant AML models, single agent Didox treatment resulted in a significant reduction in leukemia burden and a survival benefit. Didox was well tolerated, as marrow from treated animals was morphologically indistinguishable from controls. Didox exposure at levels that impaired leukemia growth did not inhibit normal HSC engraftment. In summary, Didox was well tolerated and effective against preclinical models of AML.

## Introduction

Acute Myeloid Leukemia (AML) is an aggressive, genetically heterogeneous malignancy of the marrow wherein neoplastic myeloid progenitors suppress healthy HSCs leading to marrow failure, and ultimately death. Each year in the US there are approximately 12,000 new cases and 9,000 deaths from AML [1]. This malignancy has a dismal overall five year survival rate of 30–40%, but for those over 60 overall survival drops to less than 10% [2]–[4]. AML is a disease of the elderly, with a median onset age of 70 and more than 70% of patients are over the age of 60 at diagnosis [2]. For this population the incidence of AML has slowly been climbing over the past several decades; however, the one year survival rate remains virtually unchanged [5]. These patients desperately need new treatment strategies.

The standard treatment of AML has remained unchanged for decades despite intense research [6], [7]. For those patients fortunate enough to achieve a remission most will relapse, often with chemoresistant disease [8]. Many frail and elderly patients are not candidates for additional intensive chemotherapy [9]. This highlights the need for the development of new therapeutic targets.

AML is genetically heterogenous with several distinct recurring genetic abnormalities [10]. In the last decade there have been many advances in understanding the different driving mutations in this disease. Despite this increased understanding therapies designed to target these mutations have led to only transient responses as genetically distinct subclones with decreased reliance on the target are selected for and relapse occurs. An alternative approach would be to target a “final common pathway” (i.e. a pathway that all leukemia cells, regardless of driving mutations, will need to accomplish in order to generate additional leukemia cells). One such pathway is DNA synthesis. Ribonucleotide Reductase (RR) catalyses the rate limiting step in DNA synthesis converting ribonucleotides into deoxyribonucleotides. Hydroxyurea (HU), a RR catalytic subunit inhibitor, has clinical activity in AML as a cytoreductive agent and in the palliative setting where other agents have been deemed too intensive [11]. Its effectiveness is hindered by a low affinity for RR as well as gastro-intestinal and myelosuppressive toxicities. Clinical trials in elderly and unfit AML patients have shown that HU treatment has a minimal marrow response rate [11]. Since HU has limited clinical activity in AML, RR has been an underutilized target in AML treatment. Recently, there has been a resurgence of interest in RR as a target in AML. RR has been identified as a target of 5-azacitidine, an azanucleoside used to treat AML and myelodysplastic syndromes [12]. Additionally, a phase I trial of an 20-mer antisense oligonucleotide targeting RR combined with high dose cytarabine led to a number of complete remissions in a group of poor risk patients [13]. These studies suggest that RR is a valuable target for AML treatment.

Didox is a RR inhibitor developed from HU. It has replaced the amino group with 3, 4-dihydroxyphenol. Didox displays a 20 fold more potent inhibition of RR than HU [14]. Additionally, Didox reduces both purine and pyrimidine nucleotide pools compared to purine only inhibition seen with HU [14]. Previous groups have shown Didox to have a favorable toxicity in various preclinical models compared to HU [15]–[17]. A phase I trial in metastatic carcinoma determined the maximum tolerated dose (MTD) of 6 g/m^2^ with peak plasma levels of 300 µM [18]. Didox has been shown to have activity against two AML cell lines *in vitro* with significant variability [19]. However, the efficacy of Didox in AML has not been extensively evaluated. In these studies we have examined the cellular effects and efficacy of Didox in preclinical models of AML.

## Materials and Methods

All primary samples were collected under an IRB approved protocol by Stony Brook University Medical Center or the Comprehensive Cancer Center of Wake Forest University. All patients gave written consent using an IRB approved consent form. Primary samples were obtained during clinically indicated procedures. The Comprehensive Cancer Center of Wake Forest University Institutional Animal Care and Use Committee approved all mouse experiments.

### Reagents

Didox was a gift from Howard Elford, Ph.D. at Molecules for Health, Inc. (Richmond, VA). Didox for animal studies was freshly made each time by dissolving in 5% dextrose water, with the animals receiving 425 mg/kg. For the *in vitro* studies Didox was dissolved in phosphate buffered saline (PBS) at concentrations of 10 mM and 1 mM and stored at −20°C until use. It was then diluted in the culture medium to the final concentration.

### Cell Culture Aad Viability Assays

Human lines were maintained in RPMI media (Gibco) supplemented with 10% FBS, penicillin and streptomycin. All murine lines were derived from fetal liver cells infected with MLL-ENL and NRas^G12D^ or Flt3 ITD expressing vectors [20]. Murine lines were maintained in stem cell media (40% IMDM, 40% DMEM, 20% FBS, with or without murine SCF 10 ng/mL, murine IL-6 2 ng/mL, and murine IL-3 0.4 ng/mL). Viability assays were carried out according to the manufacturer's protocols with the Cell Titer-Glo system (Promega).

### Primary Samples

All primary samples were collected under an IRB approved protocol by Stony Brook University Medical Center or the Comprehensive Cancer Center of Wake Forest University. Primary samples were obtained during clinical procedures. Cells were collected by centrifugation, resuspended in ACK lysis buffer (150 mM NH_4_Cl, 10 mM KHCO_3_, 0.1 mM EDTA) at room temperature for 5 minutes, centrifuged again, washed with PBS, and stored at −80°C until use. Normal hematopoietic stem cells were obtained from healthy allogeneic stem cell transplant donors. As an alternate method, cells were obtained by Ficoll-gradient centrifugation, and stored at −80°C until use.

### H2AX Assays

Cells were fixed in 4% neutral buffered formalin, permeabilised in PBS with 0.2% Triton-X 100. To visualize phosphorylated γH2AX, we used anti-pH2AX (#2577, 1∶100; Cell Signalling Technologies) with an Alexa Fluor 594-conjugated donkey anti-rabbit antibody (1∶1000, A-21207; Invitrogen). Cells were visualized with fluorescence microscopy.

### Western Blot

Cells were lysed in Laemmli buffer(1.6 mL 10% SDS, 500 µL 1 M Tris-HCl [pH 6.8], 800 µL glycerol, 400 2-mercaptoethanol, 4.7 mL H_2_O), and samples separated by SDS-PAGE before transfer to an Immobilon polyvinylidene difluoride membrane (Millipore). Primary antibodies against p53 (IMX25, 1∶1000; Leica Microsystems), actin (AC-15, 1∶5000; Abcam), anti-pH2AX (#2577, 1∶1000; Cell Signaling Technologies) and a secondary antibody anti-mouse (#7076, 1∶5000; Cell Signaling) or anti-rabbit (#7074, 1∶5000; Cell Signaling) were used. For RR detection, a primary antibody against the M2 subunit of RR (1∶1000; sc-10846, Santa Cruz Biotechnology) was used followed by secondary anti-goat antibody (1∶1000; ab98826, AbCam).

### Annexin V/PI Assays

Human and murine cells were plated at 100,000 cells/mL and 50,000 cells/mL respectively and treated with the indicated drugs for 48 or 72 hours. The cells were then washed in PBS and stained with propidium iodide (PI) (Sigma-Aldrich) and allophycocyanin (APC)-conjugated annexin V in a binding buffer (0.1 M HEPES [pH 7.4], 1.4 NaCl, and 25 nM CaCl2 solution; BD PharMingen) according the manufacturer's protocol. All flow cytometric analysis was carried out on the BD Accuri C6 cytometer (BD Biosciences).

### Colony Formation Studies

Primary patient samples and normal human hematopoietic stem cells (HSCs) were thawed and incubated in hematopoietic progenitor media (C-28020, PromoCell, Heidelberg, Germany) for 24 hours with a titration of Didox. Human lines were incubated as indicated above for 24 hours with a titration of Didox. Cells were washed with PBS and resuspended in IMDM supplemented with 20% FBS, and placed in ColonyGel High Cytokine Formulation media (ReachBio). Experiments were performed in triplicate. Colonies were counted on or after day 7. Colonies of 8 or more cells were counted as established in Shankar et al. [21].

### *In vivo* Efficacy Studies

The Comprehensive Cancer Center of Wake Forest University Institutional Animal Care and Use Committee approved all mouse experiments. Luciferase-tagged leukemia cells were transplanted into 8- week old, sublethally irradiated (4.5 Gy) C57Bl/6 mice by tail vein injection of 1.0×10^6^ cells per mouse. Mice were injected with 150 mg/kg D-Luciferin (Gold Biotechnology), anesthetised with isoflurane, and imaged using the IVIS 100 imaging system (Caliper LifeSciences). Mice began treatment with Didox upon detection of clear signal. The animals were treated with daily administrations of Didox at 425 mg/kg Didox (Molecules for Health) by intraperitoneal injection (IP) for 5 days. Control animals received 5% dextrose water by IP injection. Repeat imaging was performed on the day following the final treatment.

### Toxicology and Murine BM Engraftment Studies

Normal, age-matched C57Bl/6 mice were given an identical treatment regimen as the efficacy studies. Seventy-two hours following the last dose, the animals were sacrificed, bilateral femur cells harvested, and organs fixed in 10% neutral-buffered formalin followed by routine tissue processing and sectioning, and hematoxylin and eosin staining. In a blinded analysis, a veterinary pathologist reviewed the slides with a Nikon Eclipse 50i light microscope. Photographs of the tissue sections were taken with a NIS Elements D3.10 camera and software system. For the transplant assay, Ly5.1+ C57/Bl6 mice received 8 Gy of irradiation and injected with 1.0×10^6^ Ly5.2+ bone marrow cells from the Didox or control treated donors by tail vein injection. Three weeks post injection the mice were sacrificed, and bilateral femur cells harvested. The cells were stained with APC-conjugated anti-Ly5.2 Ab (BD PharMingen) and analyzed by flow cytometry.

### Statistical Analysis

Groups of 3 or more were analyzed using a one way ANOVA. All means were compared by a student's 2-tailed t test. The *in vivo* survival graphs were generated with the Kaplan-Meier method, with p values determined by the log-rank test. All analyses were performed using GraphPad Prism Version 5.02 (GraphPad Software). A p value ≤0.05 was considered significant.

## Results

### Didox is active against AML *in vitro*

**Figure 1:**
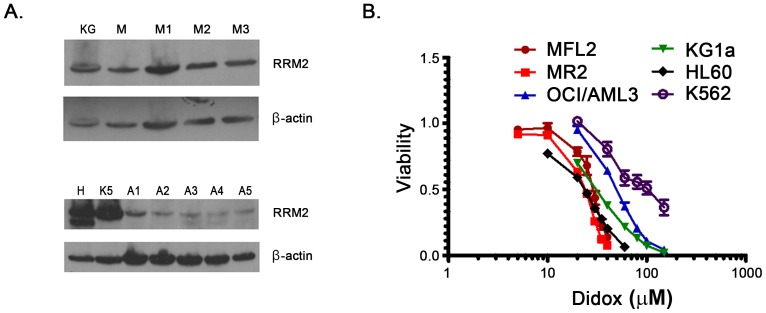
RR is expressed in AML. A. Western blots performed for RR small subunit. AML patient samples (bone marrow, M1–M3, leukopheresis A1–A5), and cell lines (K – KG1a, M – MFL2, H – HL-60, K5 – K562). B. Growth curves. Cell lines were treated with Didox for 72 hours. Viability was assessed and normalized to untreated controls.

RR has previously been shown to be upregulated in a variety of malignancies. To confirm expression of RR in AML we subjected cell lines and patient samples to western blot using an anti-RR antibody (clinical data from patient samples is shown in Table 1). Despite multiple distinct genetic abnormalities in the patient samples and cell lines we found detectable levels of RR in all samples tested (Figure 1A) consistent with RR being a final common pathway target. Having confirmed expression we sought to determine the activity of Didox in AML. We performed 72 hour viability assays with titrations of Didox (0–200 µM) in a panel of human and murine AML lines. Didox was active against all lines tested, with IC_50_'s in the low micromolar range (mean 37 µM [range 25.89–52.70 µM], Figure 1B, Table 2). These data demonstrate ubiquitous expression of RR in AML and that Didox has activity against AML cell lines at clinically achievable concentrations.

**Table 1:** Primary patient sample characteristics.

Patient	Sex	Age	Diagnosis	Karyotype
A1	M	33	AML	Del 7, inv 3
A2	M	53	AML	Normal, Flt3+
A3	F	89	AML	Trisomy 8, der(4)add(4)
A4	F	59	AML	Normal, Flt3-
A5	M	69	AML	Normal, Flt3+
C1	M	74	AML	Del 7q
C2	F	82	AML	Not obtained
C3	M	66	AML w/monocytic differentiation	Complex karyotype:
				trisomy 13, trisomy 19, t(11; 19)
M1	F	60	Acute monocytic leukemia	Trisomy 8, trisomy 9
M2	F	68	AML	Normal
M3	F	80	AML	Normal

**Table 2:** Inhibitory concentrations of murine and human AML.

Cell Line	IC_50_ (µM)	CI 95%
OCI/AML3	49.26	46.56, 52.10
KG1a	32.45	30.26, 34.79
HL-60	30.83	23.51, 40.43
K562	52.70	39.91, 69.59
MFL2	30.85	24.10, 39.49
MR2	25.89	23.75, 28.23

### Didox has activity against primary AML samples

**Figure 2:**
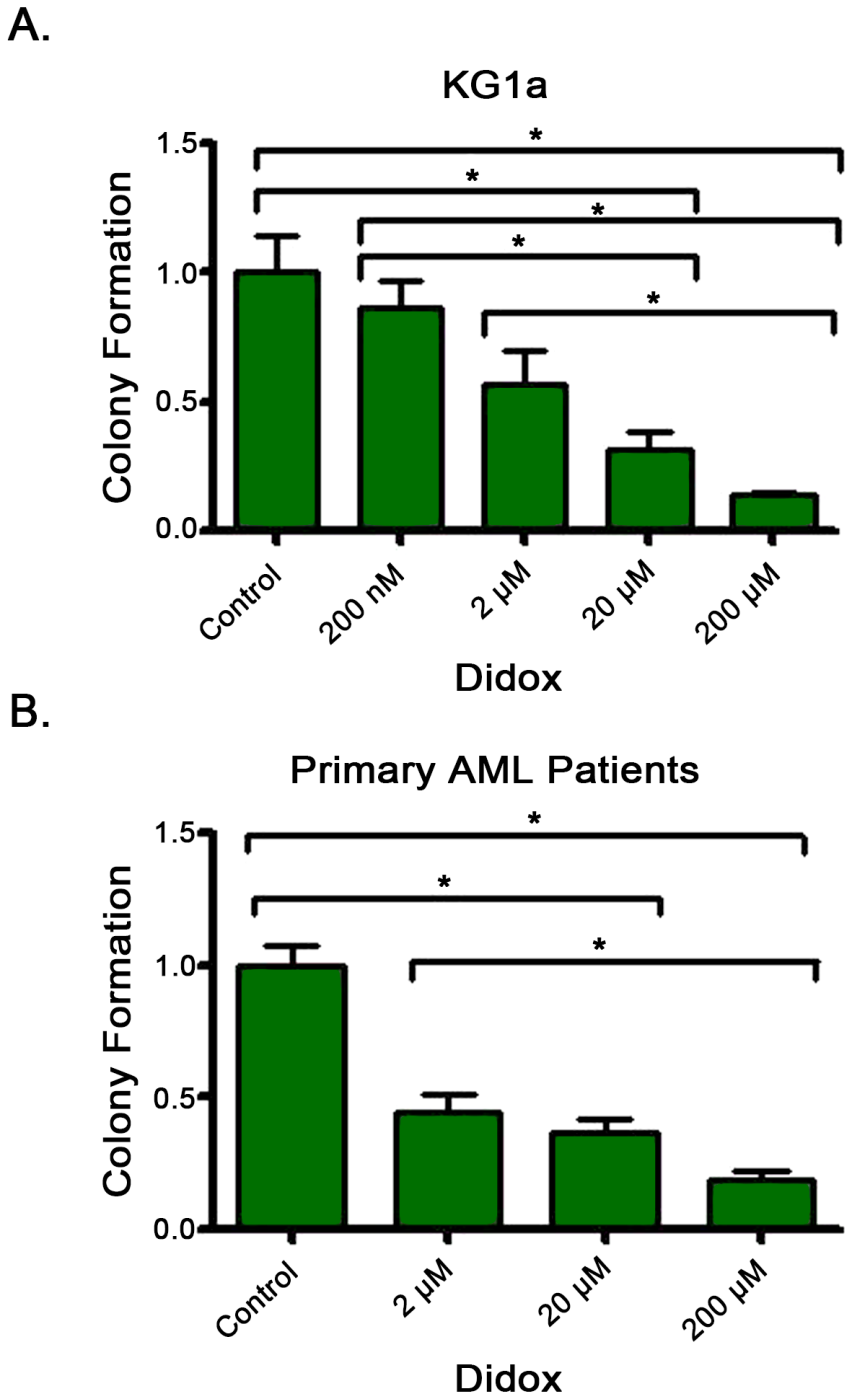
Didox has activity against AML in colony formation assays. A. Didox reduced colony formation in KG1a cells. Cells were exposed to titration of Didox for 24 hours before incubation in methylcellulose (7–8 days). Mean colony formation was assessed in triplicate in 3 experiments and normalized to untreated controls. B. Didox exposure reduced colony formation in primary AML samples. Primary samples (C1–C3) were exposed to a titration of Didox for 24 hours before incubation in methylcellulose (12–14 days). Mean colony formation was assessed in triplicate in 3 experiments and normalized to untreated controls. *  = p value less than 0.05.

As cell lines represent only a small subset of AML patients and have been kept in culture for many years, we sought to determine if Didox had any activity against primary patient samples. We performed colony formation assays on 3 primary AML samples, as well as KG1a cells. Cells were exposed to clinically achievable concentrations of Didox (0–200 µM) for 24 hours before incubation in methylcellulose (7–14 days). Consistent with our cell line data Didox, in a dose dependent fashion, significantly reduced colony formation in all samples tested (Figure 2 A–B). Didox demonstrated activity against colony forming progenitor cells from both primary patient samples and cell lines.

### Didox induces DNA damage and apoptosis

**Figure 3:**
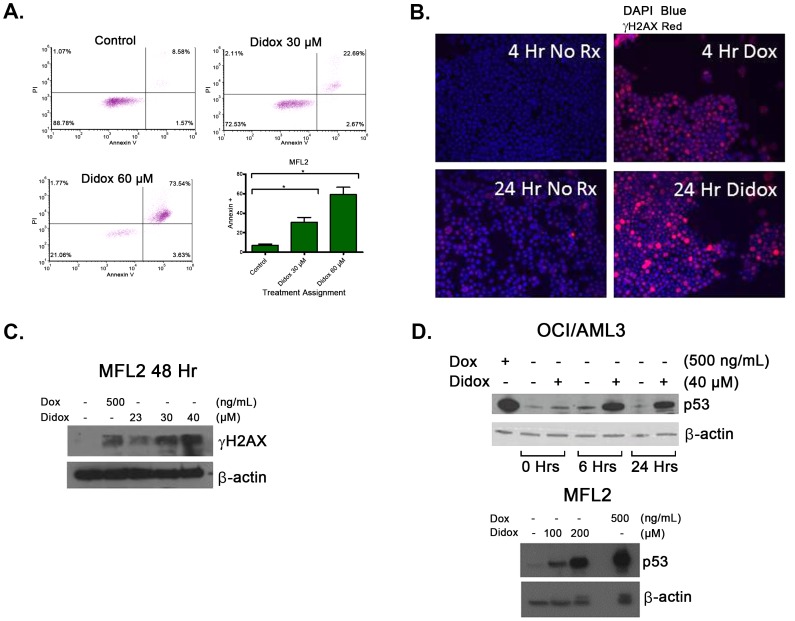
Didox induces DNA damage and apoptosis *in vitro*. A. Didox induced apoptosis at 48 hours. MFL2 cells were exposed to 30 µM, or 60 µM Didox, or a vehicle control and assessed for annexin V binding and PI staining by flow cytometry. B. KG1a cells were exposed to 20 µM Didox for 24 hours or 500 ng/mL doxorubicin for 4 hours and evaluated for γH2AX staining. C. MFL2 cells were exposed to the indicated drug for 48 hours. The cells were collected and lysed before western blot assessment for γH2AX. D. OCI/AML3 cells were exposed to Didox for 48 hours. MFL2 cells were exposed to Didox for 6 hours. The cells were collected and lysed before western blot assessment for p53. Doxorubicin at 500 ng/ml was used as a positive control. *  = p of value less than 0.05.

Previously, Didox has been shown to induce cell death via apoptosis [19], [22]. In order to determine if this occurred in our models we exposed a murine AML cell line expressing the MLL-ENL fusion protein and an internal tandem duplication mutation in the Flt3 receptor (MFL2) to a titration of Didox (0–60 µM) and collected samples at 48 hours. Samples were assessed for annexin V binding and PI staining. Didox exposure led to apoptosis in a dose dependent fashion (Figure 3A). Didox exposure results of a depletion of deoxyribonucleotides (dNTPs) leading to double strand breaks in the DNA [14]. To assess for the induction of DNA strand breaks we probed for γH2AX foci in KG1a cells exposed to increasing concentrations of Didox for 24 hours. We found an increase in positive foci with Didox exposure (Figure 3B). To confirm the induction of DNA damage we exposed MFL2 cells to a titration of Didox and performed a western blot for γH2AX. Consitent with our previous result we saw a dose dependent increase in γH2AX (Figure 3C). To evaluate the effect of Didox on DNA damage response proteins we examined p53 induction. We used the p53 sufficient cell line, OCI/AML3, which recapitulates the p53 status most often seen in AML patients [23]. Didox exposure resulted in increased p53 levels over a 24 hour period (Figure 3D). These experiments have shown that Didox exposure leads to DNA damage and subsequent p53 response, ultimately culminating in apoptosis *in vitro*.

### Didox acts through the p53 damage response pathway in p53 sufficient AMLs *in vitro*

**Figure 4:**
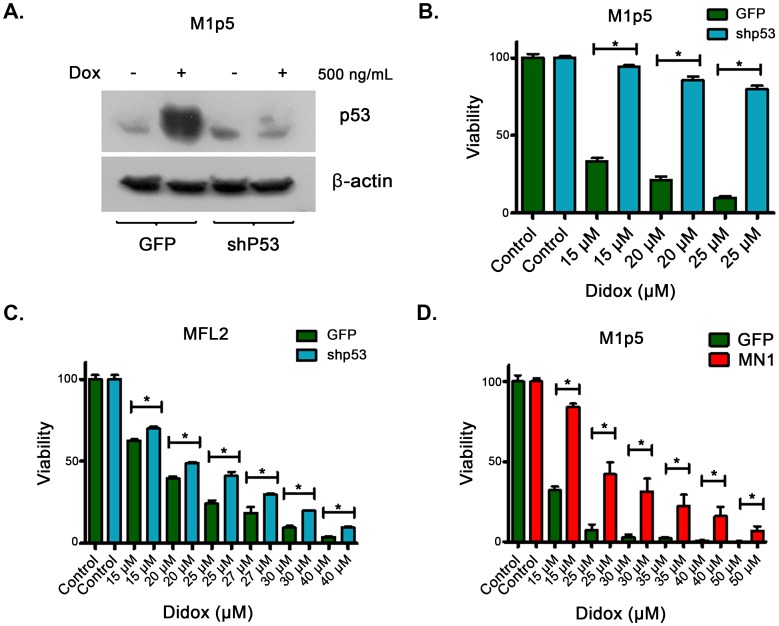
MN1 overexpression and p53 knockdown induce resistance in AML *in vitro*. A. Confirmation of KD in M1p5 cells. B. M1p5 shP53 or GFP cells were exposed to a titration of Didox (0–25 µM) for 72 hours and viability assays performed. C. MFL2 shP53 or GFP cells were exposed to a titration of Didox (0–40 µM) for 72 hours and viability assays performed. D. 3 independent 72 hour viability assays with MN1 and GFP controls, in triplicate with titrations of Didox (0–50 µM).*  = p of value less than 0.05.

In AML, p53 mutations affect 10–15% of patients leading to chemoresistance and overall poorer prognosis [24]. Given this clinical relevance and the above data that suggested Didox acted through p53, we next formally tested this by knocking down p53 in a murine AML by western blot (Figure 4A). We observed an increase in resistance to Didox in our p53 knock down compared to our controls in 3 independent viability experiments, each done in triplicate (Figure 4B). This resistance was confirmed in a second knock down of p53 in a separate murine AML (Figure 4C). Deletion of p53 is rare in AML; however, there are other clinically relevant alterations which lead to p53 suppression. Our lab has shown that p53 suppression occurs in meningioma-1 (MN1) overexpressing AML [25], along with decreased apoptosis, and chemoresistance [26]. MN1+ murine AML cells demonstrated resistance to Didox compared to GFP controls in 3 viability experiments, each done in triplicate (Figure 4D). This highlights the importance of patient selection in future clinical trials.

### Didox reduces leukemic burden and provides a survival benefit in chemoresistant models of AML *in vivo.*

In order to evaluate Didox in a more clinically relevant setting, we moved to an *in vivo* model which has been shown to recapitulate many of the features of human AML [20]. This syngeneic model has genetic lesions associated with human disease and displays many of the histopathologic features of human AML. Additionally, as an immune competent, syngeneic model, it recapitulates important immune and microenvironment interactions.

**Figure 5:**
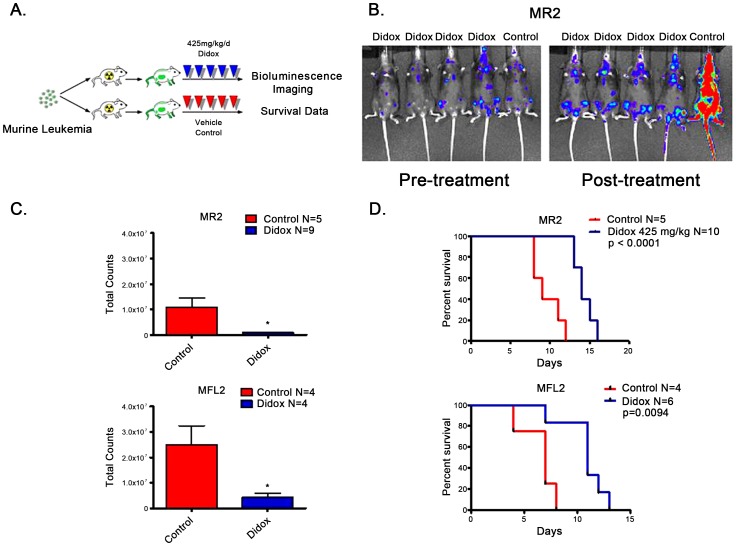
Didox has activity in AML models *in vivo*. A. Schema. 1.0×10^6^ luciferase tagged AML cells were injected into sublethally irradiated (4.5 Gy) recipients and allowed to engraft. Engraftment was monitored by bioluminescent imaging (IVIS 100 imager). Animals received 5 days of Didox at 425 mg/kg or D5 water control via intraperitoneal injection (IP). Animals were followed for survival. B. Representative bioluminescent images from Nras^G12D^ (MR2) mice pre- and post-treatment. C. Quantitation of bioluminescence post-treatment. D. Kaplan-Meier survival curves of Didox *in vivo* studies from start of treatment. *  = p of value less than 0.05.

Both *in vivo* models express the poor prognostic fusion protein MLL-ENL. The second genetic alteration needed for leukemogenesis was provided by either the Nras^G12D^ (MR2) or the Flt3 internal tandem duplication (Flt3 ITD). Luciferase tagged AML cells were injected into sublethally irradiated (4.5 Gy) recipients and allowed to engraft. Once engraftment was established by bioluminescent imaging, the animals received daily administrations of Didox at 425 mg/kg via IP injection (Figure 5A) over 5 days. Didox treatment significantly reduced leukemic burden compared to vehicle treated controls (Figure 5 B–C, p = 0.0026 and p = 0.0342). More importantly, Didox provided a significant survival benefit (Figure 5D, p<0.0001 and p = 0.0094). This data demonstrates that Didox has activity against syngeneic AML models *in vivo*.

### Didox is well tolerated in normal C57Bl/6 mice, and does not harm hematopoietic stem cells

**Figure 6:**
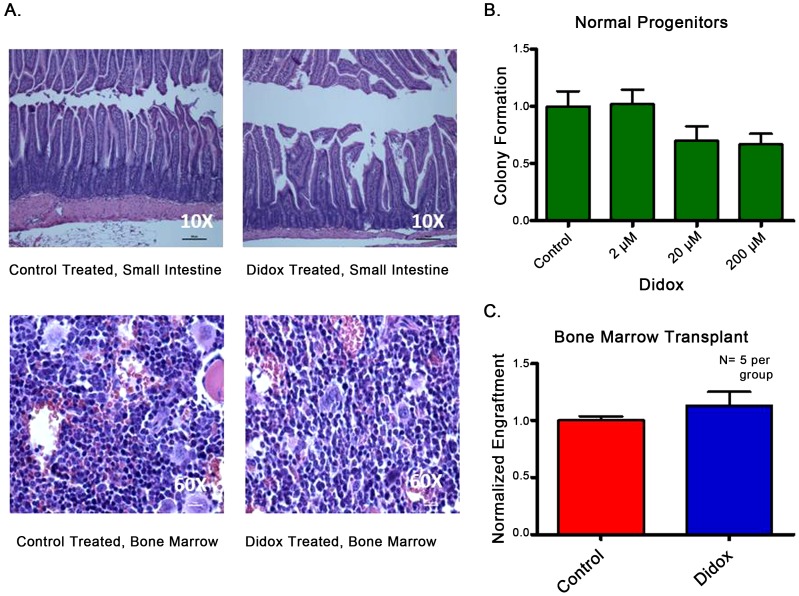
Didox is well tolerated. A. Didox treated C57Bl/6 mice showed no difference in tissue morphology compared to vehicle treated controls as read by a veterinary pathologist blinded to treatment assignment. Representative H&E sections of gastrointestinal tract (Small Intestine) and bone marrow from Didox (n = 3) and control treated animals (n = 3). B. Colony formation assays performed on normal HSCs following 24 hour Didox exposure (0–200 µM), p = 0.09. C. Didox treatment does not harm normal HSCs. C57Bl/6 mice were treated for 5 days with 425 mg/kg Didox or a vehicle control via IP injection. 72 hours post treatment the animals were sacrificed and their marrow harvested. Marrow was then transplanted into lethally irradiated (8 Gy) Ly5.1+ recipients and allowed to engraft. Post-engraftment the animals were sacrificed, marrow harvested, and analyzed for Ly5.2+ by flow cytometry. Engraftment values were normalized to vehicle controls. N = 5 per group.

Since we have shown that Didox treatment reduced leukaemic burden compared to controls *in vivo*, we wanted to interrogate its effects on normal tissues at the dose and schedule used in the survival studies. Normal C57Bl/6 mice received the same Didox regimen as the efficacy study mice and were sacrificed 72 hours following the final treatment. In a blinded analysis, a veterinary pathologist was unable to distinguish morphological differences between the two groups (Figure 6A). This demonstrates that Didox has minimal effect on normal tissue morphology. However, this does not tell us the consequences of Didox treatment on the function of normal HSCs. To determine the effects of Didox on normal human hematopoietic progenitors we performed colony formation assays on 3 normal samples. In contrast to our results with primary patient samples Didox treatment lead to only a modest and non-significant reduction in colony formation of normal progenitors, even at the highest dose tested (Figure 6B). In order to determine the effect of Didox on normal HSCs we determined the ability of Didox treated marrow cells to engraft in syngeneic recipients. Normal C57Bl/6 mice (Ly5.2+) were treated as in the AML efficacy studies and their marrow harvested 72 hours following last treatment and transplanted into lethally irradiated Ly5.1+ recipients. After 3 weeks recipients were sacrificed and engraftment was determined by flow cytometry (Figure S1). Didox treated marrow engrafted at least as well as the control marrow (Figure 6C). These data demonstrate that Didox does not cause gross tissue toxicity at the effective dose in C57Bl/6 mice, nor does it harm the function of normal progenitors or HSCs. These data suggest a large therapeutic window.

## Discussion

AML is an aggressive malignancy that primarily effects the elderly population. It is characterised by high genetic heterogeneity and poor overall 5 year survival [2]. The frontline treatments in AML have remained virtually unchanged for decades, and while many patients may have a transient response to chemotherapy, most will relapse with chemoresistant disease [8]. This highlights both the dearth of progress in AML treatment and the desperate need for the development of new therapies.

A strategy that targets a metabolic pathway required by all leukemia cells regardless of driving mutation has the potential to be effective even in a genetically heterogenous disease like AML. One such pathway is DNA synthesis. The rate limiting reaction of DNA synthesis is catalysed by RR and has been shown to be upregulated in many malignancies [27]–[30]. The classical inhibitor, HU, has had limited use in the clinic due to poor affinity to RR, lack of durable responses and associated toxicities. However, there has been a resurgence of interest in RR inhibition in AML.

Didox was developed from HU and displays 20 fold more potent affinity for RR than its predecessor. It reduces both purine and pyrimidine pools. Moreover, it has been shown to have a more favorable toxicity profile compared to HU in preclinical models [15], [31]. The MTD was determined from a phase I trial, but it has not yet been extensively studied in AML.

We have investigated the efficacy of Didox, a novel RR inhibitor, *in vitro* and *in vivo* in preclinical models of AML. We made several key observations: 1. RR was ubiquitously expressed in all samples and cell lines tested. 2. Didox had activity in all cell lines and patient samples tested with IC_50_ values in the low micromolar range. 3. Didox exposure led to DNA damage, p53 induction, and apoptosis. 4. Didox was effective against two *in vivo* models of AML. 5. Didox treatment did not cause gross tissue toxicity in non-leukemic animals. And finally, Didox did not harm normal haematopoietic progenitors or stem cells.

Didox had activity across a panel of cell lines and primary patient samples with diverse cytogenetic characteristics, suggesting inhibition of RR is effective regardless of their driving mutations. This is supported by our finding that RR is expressed in all cell lines and patient samples. The IC_50_ values for all lines tested clustered in the low micromolar range with a mean value of 37 µM (range 25.89–52.70 µM) despite the wide variety of driving mutations in the lines tested. Importantly, all IC_50_ values were well below the peak plasma levels achieved at the MTD of Didox in a phase I clinical trial [18]. In addition, primary patient samples were also impaired in their ability to form colonies following Didox exposure at levels below those achieved in clinical trials. This is the first data, to our knowledge, that demonstrates Didox efficacy against primary patient derived AML cells. These results suggest that Didox is effective at doses that are achievable in a clinical setting.

Didox has been shown to cause reductions and imbalances in the dNTP pools in multiple cancer cell lines including leukaemia cells [19], [32]. This dNTP imbalance can lead to several consequences including nucleotide misincorporation and stalled replication forks (reviewed in [33]). Didox treatment also suppresses RAD51 expression, a key DNA repair enzyme in myeloma cells [22] and inhibits the upregulation of other DNA repair proteins in gliosarcoma cells [32]. This simultaneous induction of DNA damage and inhibition of repair results in apoptosis. This mechanism is attractive for the treatment of AML as patient samples have shown impairments in DNA damage response [34]. Consistent with this we have demonstrated Didox induces DNA damage and increased p53 levels followed by apoptosis in our models.

In previous studies, this laboratory has examined the effects of MN1 in AML. MN1 overexpression is associated with a poor prognosis in patients. Its overexpression led to accelerated leukemic growth, chemoresistance, suppression of p53, and decreased apoptosis in preclinical models. This increase in resistance seen with MN1 overexpression may be due to the previously described p53 suppression in these cells.

Our *in vitro* results demonstrate that Didox, when present throughout a 24 or 72 hour period at clinically achievable concentrations efficiently induce leukemia cell death. However, they do not address the ability Didox to induce leukemia cell death when given as a daily bolus with leukemia cells in their appropriate microenvironment. Several studies have demonstrated the protective effect of the marrow microenvironment in AML [35]–[37]. Our *in vivo* studies using a syngeneic, immunocompetent AML model demonstrate a reduction in leukemic burden and a significant increase in survival following 5 daily doses of Didox. These data show that Didox can induce leukemia cell death even in the marrow microenvironment and further suggest it will be an effective agent in the treatment of AML patients.

In previous reports Didox has been shown to be less toxic to the hematopoietic system than HU [38]. Suppression of normal hematopoiesis by current AML therapies is a major cause of treatment related mortality in these patients. Our studies have confirmed the low toxicity of Didox on normal hematopoietic progenitors *in vitro* and for the first time on HSCs *in vivo*. The reasons for this large therapeutic window are not clear, but there are several possible contributing factors. Leukemia cells are likely to have a high reliance on RR for proliferation as RR activity has been shown to correlate with proliferation and to be elevated in cancer cells [39]. Furthermore, oncogenic transformation is an inherently stressful process and renders cells more susceptible to DNA damage [40].

In summary, our results highlight an underutilized target in AML treatment through the use of a novel inhibitor. We demonstrated the activity of Didox both *in vitro* and *in vivo* in preclinical models of AML. Consistent with previous studies in other models Didox was well tolerated, with limited toxicities, suggesting that this is a promising therapeutic for combination regimens with both targeted and standard therapies [15], [31]. Such studies are currently underway.

## Supporting Information

Figure S1 **Facs analysis of engrafted Didox treated marrow.** Shown is a representative dot plot and histogram analysis of femur samples collected from Ly5.1+ C57Bl/6 mice following injection with Didox treated Ly5.2 treated marrow cells.
(TIF)
