# Supplementary figures and images for: The Efficacy of the Ribonucleotide Reductase Inhibitor Didox in Preclinical Models of AML
Source: PLoS One. 2014 Nov 17;9(11):e112619. doi: 10.1371/journal.pone.0112619 (PMC4234372; doi:10.1371/journal.pone.0112619)

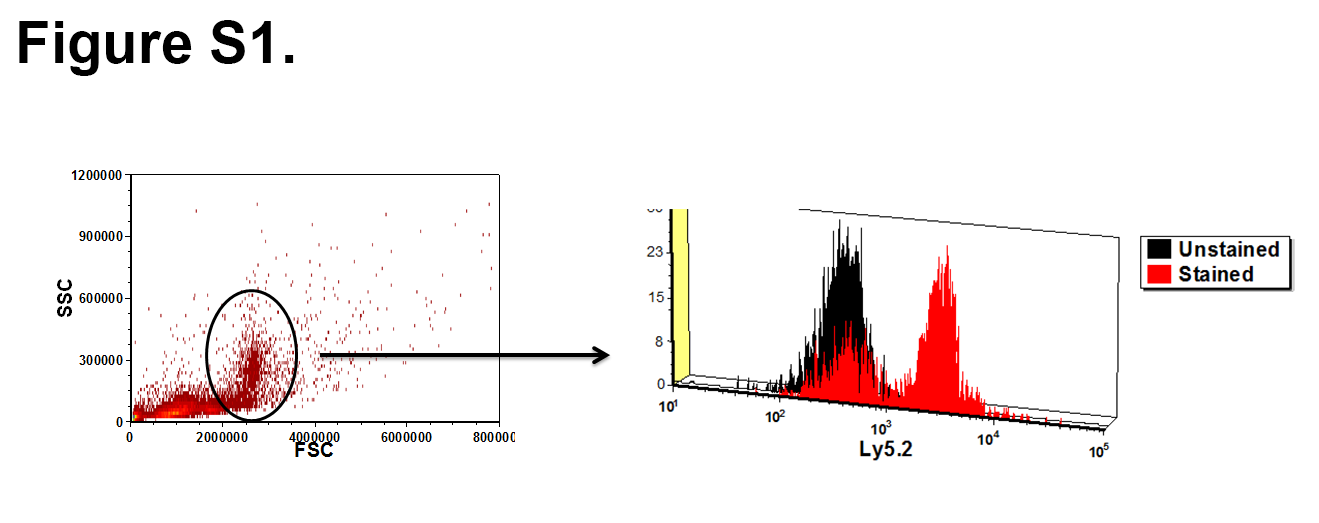

Supplement: Figure S1 — Facs analysis of engrafted Didox treated marrow. Shown is a representative dot plot and histogram analysis of femur samples collected from Ly5.1+ C57Bl/6 mice following injection with Didox treated Ly5.2 treated marrow cells. (TIF) [file pone.0112619.s001.tif]
